# “Horchata” drink in Southern Ecuador: medicinal plants and people’s wellbeing

**DOI:** 10.1186/s13002-017-0145-z

**Published:** 2017-03-09

**Authors:** Montserrat Rios, Fani Tinitana, Pablo Jarrín-V, Natalia Donoso, Juan Carlos Romero-Benavides

**Affiliations:** 1 Departamento de Química y Ciencias Exactas, Universidad Técnica Particular de Loja, San Cayetano Alto, Loja, Ecuador; 2 Institute for Tropical Ecology and Conservation, University of Florida, Gainesville, FL 32611-0430 USA; 3 Departamento de Ciencias Biológicas, Universidad Técnica Particular de Loja, Loja, Ecuador; 4 Universidad Regional Amazónica Ikiam, Tena, Ecuador

**Keywords:** Horchata drink, Herbal mixture, Medicinal plants, Traditional markets, Fidelity level, Factor of informant consensus, Cluster analysis, Loja province

## Abstract

**Background:**

The “horchata” is a herbal mixture infusion consumed in Southern Ecuador. It remains unknown how vendors group the plant species to sell them at traditional markets. This research documented the following: 1) a list of medicinal plant species sold for the drink; 2) the culturally important medicinal plant species; 3) the agreement among vendors regarding the medicinal plants species and their therapeutic use; and 4) the groups of medicinal plants sold for the preparation of “horchata.”

**Methods:**

Interviews were made to 185 vendors at 31 traditional markets in Loja province. Bunches of medicinal plants were purchased to identify the species and to prepare voucher specimens. Culturally important medicinal plants species were established with the Fidelity Level (FL) index. Agreement among vendors on the therapeutic use of medicinal plants was measured with the Factor of Informant Consensus (FIC) index. A cluster analysis was made to determine the groups of medicinal plants sold by market vendors to prepare the “horchata” drink.

**Results:**

In Loja province, the “horchata” drink is consumed for its therapeutic uses. This study registered 33 families with 58 genera and 71 medicinal plant species, 50 of which are herbs and three are endemic to the Andean highlands of Ecuador. The FL index (46.1–96.3) determined 20 culturally important medicinal plant species. The highest FIC value (1.00) among vendors corresponds to four plant species employed each for a different therapeutic use. The cluster analysis identified a core group of 16 plant species which are essential to the drink and which likely interact to provide wellbeing.

**Conclusions:**

The “horchata” is a heritage drink in Loja province. The 71 medicinal plants species registered for this drink is the largest number reported to date, and they have a total of 32 therapeutic uses. The combined results of the FL and FIC indices, the cluster analysis, and the field observations reveal an agreement among vendors on 16 medicinal plant species and their therapeutic use. This core group of plants requires bioactivity and bioassays analyses to determine biomedicine benefits that would be based on their pharmacological properties.

## Background

The chronicled documentation on the history of “horchata” consumption is quite ancient, as it outlines the existence of a beverage made with the tuber of “chufa” or earth almond (*Cyperus esculentus* L.) that was drunk in early Egypt (2400 B.C.). There is evidence of this drink being found in vessels, vestiges of certain Pharaoh’s tombs [1–3] and in the Ebers Papyrus [4]. This culturally valuable drink was also ingested in South Sudan, especially in a region named Chut, where is the African origin of *Cyperus esculentus* L. [2, 3, 5–7]. In this region, this plant is commonly known as nut sedge or yellow nut grass, and is locally named in Arabic as “hab elaziz.” With the conquest of Egypt by the Roman Empire (30 B.C.), the Romans introduced this drink to their culture and named it in the Latin voice “hordeāta”, “hordiate” or “orzata” [8].

In the early 700’s, after the conquest of Southern Spain, Moorish traders introduced the cultivation of the “chufa” plant in the Mediterranean region [9–11]. Evidence of the introduction of this cultivar has been found in the province of Valencia, where its sandy land and mild weather is favorable to the “chufa” as a weedy cultivar [9–12]. Local people in this region of Spain consumed the extract of the “chufa” tuber and enjoyed the resulting beverage [9–12]. Historical records reveal that the Latin voice “hordeāta” is the origin for the Spanish term “horchata” that appeared in the 1200’s in Valencia, when this drink was offered to King Jaime I in Alboraya as “leche de chufa” (tiger nut milk) [8, 9, 13].

King Jaime I named the “horchata” drink as “oro, chata” in his original Valencian language, which has Latin roots, and became with time and variable pronunciations the expression “or, xata, xufa” and later “orchata de chufa” [9, 13]. The history of this drink in Spain reveals that it became popular with a variant prepared with barley (*Hordeum vulgare* L.) and known as “agua de cebada” (barley water) [9, 14]. These two kinds of “horchata” preparations are still consumed in coastal areas near Valencia [9, 14]. Since the Roman Empire (30 B.C.) until the present, the term “horchata” reveals how a beverage can maintain its name by historical tradition and along an extended time line, even when its ingredients adapt accordingly to the plant resources of a country and the local taste of its people.

At present-day Spain, “horchata” is a drink made of barley mixed with fruit extracts, water and sugar [14, 15]. In Latin America, “horchata” is a term used to identify a wide variety of beverages, as its ingredients are different in each country and even at particular national regions [16]. For instance, in Guatemala and Mexico “horchata” is known as “aguas frescas” and made with almonds, cinnamon, rice, vanilla, and sugar; in Venezuela, it is named as “chicha” and prepared with sesame seeds, sugar and water [16]. In Peru, similarly to Spain, there is a drink based on barley but made with toasted grains and around 42 medicinal plant species, locally known as “emoliente” [17, 18].

In Ecuador, particularly in its Southern region, “horchata” is also called “aguas frescas” or “agua de frescos” [19, 20], which is an herbal mixture infusion of 16 to 32 medicinal plants with sugar, honey or raw cane sugar and lemon drops. The “horchata” drink has existed in the Southern Andean Ecuador since the Spanish colony, and has been traditionally prepared with medicinal plants from the local production [21]. “Horchata” is very popular in the province of Loja, particularly among the indigenous people who believe that the therapeutic effect of the drink is improved when plants locally known as “calientes” (warm) and “frías” (cold) are properly combined [21]. The historical tradition of consuming the “horchata” drink persists nowadays in the Loja province, and has noticeable cultural impact in the local population due to the belief in its benefits [19, 21].

The urban and rural people who consume the “horchata” drink in the Loja province report wellbeing benefits, and believe that this herbal mixture infusion promotes a healthy digestion, improves memory, and acts as an hepatic anti-inflammatory and a diuretic [21]. Most of the local population in this region consumes “horchata” with meals, either cold or hot [17]. It is a refreshing drink of fuchsia tonality that is served at homes or sold at traditional markets [17]. In the ancestral memory of indigenous populations, especially those located in the Southern Andean highlands of the country; the intense color of “horchata” is associated with physical and spiritual strength. Through this perception, people that consume this drink feel invigorated.

Despite being frequently consumed by local people, studies on healing herbal drinks prepared with medicinal plants are scarce for the Andean region [18, 22–24], and this represents a gap in ethnobotany. This lack of knowledge is also present in Ecuador, especially regarding beverages that are typical to each region [19, 25], as is the case of “horchata,” which is mentioned in a few studies only [17, 21, 22, 26–30]. In this context, this research documents the following: 1) the list of medicinal plant species sold for the drink; 2) the culturally important medicinal plants species as determined by the Fidelity Level (FL) index; 3) the agreement among vendors regarding the therapeutic uses of medicinal plants as measured by the Factor of Informant Consensus (FIC) index; and 4) the groups of medicinal plants sold for the “horchata” drink as defined by a cluster analysis.

## Methods

### Study area

**Fig. 1:**
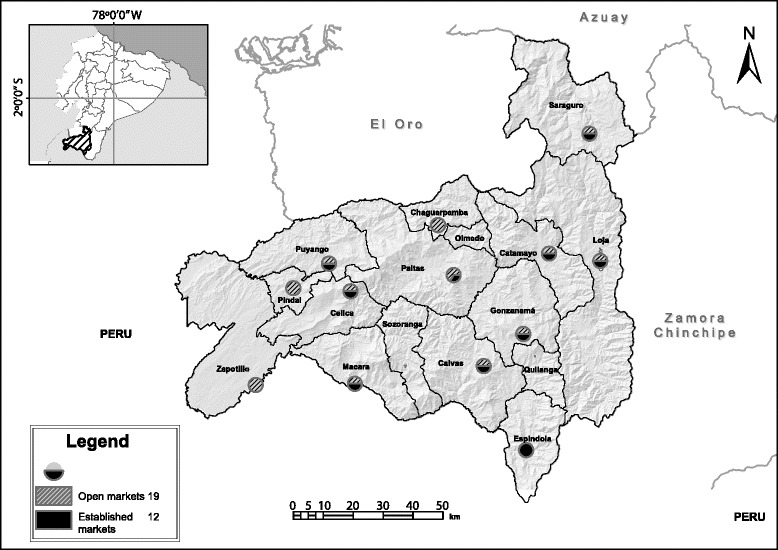
Traditional markets location in the Loja province, Southern Ecuador

The study was conducted in the Loja province where “horchata” has a significant cultural value and it is traditionally consumed. This region is located in Southern Ecuador, between 3°19’56”S to 4°44’36”S and 79°04’28”W to 80°29’03”W (Fig. 1), occupies 11.042 km^2^ that represent 4% of Ecuador’s territory, and borders to the south with Peru [31]. The total population of the province in 2010 was 448,966 inhabitants, with 96.3% corresponding to “mestizo” Spanish speakers and 3.7% to Saraguro indigenous people [32]. The latter population speaks both Spanish and Kichwa languages [32].

The Loja province has abundant hydrographic resources, such as rivers which flow either into the Pacific catchment basin or the Amazonian lowlands [33]. The province is dominated by the Andean mountain range, which gives rise to a very irregular topography with elevations ranging from 120 to 3800 m [33]. This area shows a considerable variety of local climate, with tropical dry conditions in the west, subtropical humid in the central area, and cold humid towards the east [31]. Most of the soil is ferralitic, yellow/reddish and rich in humus [33].

### Traditional markets

The Loja province has 16 political units known as “cantones” and this study was conducted at 13 which are: Calvas, Catamayo, Celica, Chaguarpamba, Espíndola, Gonzanamá, Loja, Macará, Paltas, Pindal, Puyango, Saraguro, and Zapotillo. Medicinal plants to prepare “horchata” are sold at 31 traditional markets, which are divided in 12 established markets located at 12 “cantones”, and 19 open markets located at 13 “cantones” (Fig. 1). According to the classification by Tinitana et al. [30], the established markets are situated within a permanent building, while the open markets occur weekly along nearby streets and sidewalks.

Women are often the vendors involved in the sale of medicinal plants for the preparation of “horchata”. The traceability of the medicinal plants used to prepare this herbal mixture infusion starts at those women who cultivate plants in their homegardens and ends at the final sell. Vendors negotiate whole plant individuals or plant resources to retailers, either directly to customers at open markets or to formal vendors at stalls in stablished markets. The three criteria applied to determine the kind of vendors was partially based on Tinitana et al. [30], on how they self-recognized their role, and on field observations (Table 1). This classification determined three different kinds of women vendors according to their role in the sale of medicinal plant species used to prepare “horchata” (Table 1).

**Table 1:** Kinds of medicinal plants vendors and their role in traditional markets in the Loja province

Vendor	Definition
Rural harvesters and small suppliers	Women who live in rural areas surrounding the main cities in the Loja province, and who cultivate medicinal plants growth in their homegardens or collect fresh medicinal plants in nature (Table 2). They sell at home or travel to cities to trade plant bunches to prepare “horchata”, negotiating with customers or formal vendors at open markets and/or established markets.
Formal vendors	Women who legally hold an operating license from the government to rent a stall in the established markets for trading fruits, vegetables, medicinal plants and bunches to prepare “horchata”.
Informal vendors	Women who come from rural or metropolitan areas of the Loja province, and are market vendors on foot. They are minor public resellers of bunches to prepare “horchata” at established and/or open markets.

### Data collection

Medicinal plants to prepare horchata are generally traded in a group locally named as “atado”, “manojo”, “ramillete” or “tongo” that in the present study will be defined as a “bunch” and is equivalent to the term “portfolio” in Bussman et al. [34]. Surveys on these medicinal plant bunches were conducted at the 13 “cantones” in the Loja province between 2012 and 2015. To record the most frequently sold medicinal plant species to prepare “horchata”, a total of 72 field visits were made to 31 traditional markets.

The second author carried out interviews with 185 vendors, which were divided in 56 formal vendors at stalls in established markets, and 129 informal vendors at open markets. After explaining the aim of the study to all vendors of medicinal plants from the 31 traditional markets, they were asked to participate in the research and accepted on their own will. The interviewed vendors were older than 31 years old; they were 161 mestizos (87%) and 24 Saraguro indigenous people (13%). It is remarkable that all vendors were women.

“Horchata” bunches were purchased from each vendor, and an interview was made with structured ethnobotanical questionnaires which were willingly answered without payment. The second author conducted the interviews in Spanish, and accepted the will of those vendors who preferred to remain anonymous. The aim of the questionnaires was to record information on the plant specimens, specifically data on the following aspects: medicinal uses and health benefits, local vernacular names and if the whole plant or a morphological structure was sold.

This study applied the code of ethics of the International Society for Ethnobiology (ISE) [35], also endorsed by the Society for Economic Botany (SEB), specifically the Principle of Respect, which recognizes the necessity for researchers “to respect the integrity, morality and spirituality of the culture, traditions and relationships of indigenous people, traditional societies, and local communities with their worlds”. The interviews with vendors were made under mutually agreed conditions and according to Ecuador’s rights, especially with regards to the Convention on Biological Diversity (CDB) [36].

### Nomenclature, geographic range, conservation status, and voucher collection

The nomenclature of plant families, genera and species follows the Catalogue of Vascular Plants of Ecuador [37]. It was also compared to the TROPICOS database [38] and the classification for orders and families of flowering plants proposed by The Angiosperm Phylogeny Group APG IV [39]. The 71 species recorded in this study were identified using the available volumes of the Flora of Ecuador [40–43] and reference material in the herbaria of the “Universidad Técnica Particular de Loja” (HUTPL), “Universidad Nacional de Loja” (LOJA) and “Pontificia Universidad Católica del Ecuador” (QCA).

The geographic status or distribution range for the 71 medicinal plant species was corroborated in the TROPICOS database [38], and then each species was classified as native, endemic or introduced. The conservation status was revised in the databases of TROPICOS [38], the Red List of the International Union for Conservation of Nature (IUCN) [44], and the red book of endemic plants of Ecuador [45]. The categorization system of the IUCN and the criteria of the red lists for plants establish conservation priorities and determine the degree of threat of a species in nature according to eight categories [46].

The plant specimens were registered under the collection series FT (Fani Tinitana) and vouchers were deposited at HUTPL. The collection of the plant specimens was authorized by the Ministry of Environment of Ecuador (Ministerio del Ambiente del Ecuador N° 001-2013-IC-FLO-DPAP-MAE and N° 001-2015-VS-DPL-MAE).

### Participant observation

The participant observation technique has a strategic value as it allows observing the behavior of people in situ [47–49]. Participant observation permitted establishing a correlation between women, who self-recognize as “horchateras”, and the medicinal plant species that are used in the preparation of “horchata”. The visits made by the first author to rural communities provided a direct approach to observe the role of “horchateras”, especially in the growing and caring of the medicinal plants. In the indigenous communities surrounding the cities of the Loja province, each “horchatera” has a homegarden.

During one year of field visits, “horchateras” were observed and interviewed along walks across homegardens and different areas of surrounding vegetation. These interactions allowed registering how women cultivated medicinal plant species or collected them in nature. Within this context, women are those who care for the production of the homegarden, while men work at vegetable crops, agroforestry lands, monocultures of sugarcane and fruits, cattle ranching and construction. It is noteworthy that some “horchateras” grow medicinal plants without chemical products and maintain ecological sustainable and environmentally friendly agricultural practices.

### Fidelity level index

The fidelity level (FL) index is the percentage of vendors independently claiming a medicinal use for a certain plant species [50]. The FL index was calculated for each of the 71 recorded species used to prepare “horchata” through the following formula FL (%) = (I_p_ × 100/ I_u_); where I_p_ is the number of vendors who independently cited the use of a specific medicinal plant species and I_u_ is the total number of vendors [50].

The cultural importance of each reported medicinal plant species sold to prepare “horchata” at the 31 traditional markets was evaluated with the FL index [50–52]. Accordingly, 185 women market vendors were interviewed and their answers noted. The therapeutic uses identified and recorded in the field for each plant species were interpreted, and later adapted to the categories proposed by Rios et al. [25] for the useful plants of Ecuador.

### Factor of informant consensus index

The factor of informant consensus (FIC) index is a measure of agreement among vendors on the therapeutic use of a medicinal plant species [53]. The FIC values range between 0 and 1, where 1 indicates the highest level of market vendor consensus. The value was calculated for each of the 32 therapeutic uses mentioned by the 185 vendors according to the following formula: FIC = (Nur – Nt)/(Nur – 1), where Nur is the number of vendors reporting a particular therapeutic use, and Nt is the total number of medicinal plant species used for a particular therapeutic use [53, 54].

### Cluster analysis

The aim of cluster analysis was to group and compare the different bunches sold by the vendors for “horchata” preparation. The compiled data were organized in a data matrix. Market vendors were listed as columns and medicinal plant species as rows. The cells have values of 1 (presence) or 0 (absence). The original matrix was analyzed in the statistical package Vegan v. 2.2-0 [55] to estimate: 1) a similarity matrix with the Jaccard index that weights the presence/absence data and provides a “clear direct interpretation” [56] of bunch similarity [57–61] based on medicinal plant species, 2) a cluster analysis based on the UPGMA method and its corresponding dendrogram, and 3) the cophenetic correlation coefficient (r) by comparing the original distances to the ultrametric distances in the dendrogram.

## Results and discussions

### Medicinal plant species sold for the “horchata” at traditional markets

According to the results of the taxonomical identification, the medicinal plants used to prepare the “horchata” drink belong to 71 species, grouped in 58 genera and 33 plant families (Table 2). The current study registers the largest number of medicinal plant species known so far for any herbal mixture drink in Ecuador, contrasting to 28 species reported by Arguello and Aguilar [27] for the Loja province, 60 species mentioned by Cerón [17] at 12 traditional markets in Cuenca and Loja cities, and 28 species recorded by Villamagua Vergara [21] for highlands indigenous communities in the Loja province. In the case of Peru, Bussmann et al. [18] registered 42 species for the preparation of “emoliente” drinks that are sold by “emolienteros”.

**Table 2:** Medicinal plants used to prepare “horchata” and sold at 31 traditional markets in Loja province

Scientific name	Vernacular name	Geographic status	Habit	Morphological structure used	Therapeutic use	Conservation status	Voucher number
Adoxaceae							
*Sambucus nigra* L*.*	Sauco, tilo	Introduced	Tree	Flower	Antiflu, antitussive, sedative	NE	FT1252
Amaranthaceae							
*Aerva sanguinolenta* (L.) Blume	Escancel	Introduced	Herb	Plant without root	Analgesic, anti-inflammatory, antiseptic, diuretic, emmenagogue, restorative, stomachache	NE	FTMAL008
*Alternanthera porrigens* (Jacq.) Kuntze	Moradilla	Native	Shrub	Branch, flower	Analgesic, anti-inflammatory, diuretic, restorative, tonic	NE	FT0010
*Amaranthus caudatus* L.	Amaranto, ataco, ataku morado, sangorache	Native	Herb	Inflorescence	Antiflu, anti-inflammatory, blood circulation, carminative, diuretic, emmenagogue, hepatic, stimulant, tonic	NE	FT0278
*Amaranthus hybridus* L.	Ataco, ataku, bledo, sangorache	Native	Herb	Inflorescence, young leaf	Antiflu, anti-inflammatory, diuretic, emmenagogue, tonic, vulnerary	NE	FTMAL006
*Iresine diffusa* Humb. & Bonpl. ex Willd.	Chulku, escancel	Native	Herb	Branch	Analgesic, antiflu, anti-inflammatory, diuretic, hepatic, tonic	NE	HUTPL433
*Iresine herbstii* Hook.	Escancel, tigrecillo	Native	Herb	Branch, shoot	Analgesic, antiflu, anti-inflammatory, diuretic, sedative, tonic	NE	FT0486
Apiaceae							
*Foeniculum vulgare* Mill.	Eneldo, hinojo	Introduced	Herb	Leaf	Analgesic, carminative, digestive, diuretic, hepatic, stomachache, tonic	NE	FT0025t
*Niphogeton dissecta* (Benth.) J.F. Macbr.^a^	Culantrillo	Native	Herb	Plant without root	Analgesic, carminative	NE	FT0024t
Asclepiadaceae							
*Orthosia ellemannii* (Morillo) Liede & Meve^a^	Cola de caballo	Endemic	Vine	Branch	Anti-inflammatory, diuretic	VU	FT037t
Asteraceae							
*Gamochaeta americana* (Mill.) Wedd.^a^*	Lechugilla, lancetilla	Native	Herb	Plant without root	Antiflu, antidiarrheal, vulnerary	NE	FT026t
*Matricaria recutita* L.	Manzanilla	Introduced	Herb	Plant without root	Analgesic, antidiarrheal, anti-inflammatory, carminative, digestive, sedative, stomachache	NE	FT0014t
*Sonchus oleraceus* L.	Kana yuyu, cerraja	Introduced	Herb	Plant without root	Analgesic, antiflu, anti-inflammatory, antispasmodic, diuretic, hepatic, stomachache	NE	FT28t
*Tagetes filifolia* Lag*.*	Anís	Native	Herb	Flower, leaf	Anti-inflammatory, antiflu, carminative, digestive, febrifuge, sedative, stomachache, tonic	NE	FT0987
*Taraxacum officinale* F.H. Wigg.	Diente de león	Introduced	Herb	Whole plant	Analgesic, anti-inflammatory, cholagogue, depurative, digestive, diuretic, hepatic, restorative, stomachache, vulnerary	NE	FT0029t
Begoniaceae							
*Begonia* × *tuberhybrida* Voss	Begonia	Native	Herb	Petal	Sedative, tonic	NE	FT1250
Boraginaceae							
*Borago officinalis* L.	Borraja	Introduced	Herb	Flower, leaf	Analgesic, antidiarrheal, antiflu, anti-inflammatory, antitussive, emmenagogue, febrifuge	NE	FT011MAL
Brassicaceae							
*Matthiola incana* (L.) W.T. Aiton	Alhelí	Introduced	Herb	Flower	Analgesic, antiflu, cardiotonic, digestive, hepatic, sedative, stomachache	NE	FT1250
Caryophyllaceae							
*Dianthus caryophyllus* L.	Clavel	Introduced	Herb	Petal	Analgesic, anti-inflammatory, cardiotonic, restorative, sedative	NE	FT1253
Commelinaceae							
*Callisia repens* (Jacq.) L.	Calcha, calsi	Native	Herb	Leaf	Hypotensive	NE	FT51MPAT
*Tradescantia zebrina* Heynh. ex Bosse	Calcha	Introduced	Herb	Leaf	Analgesic, febrifuge, hypotensive	NE	FT30t
Equisetaceae							
*Equisetum bogotense* Kunth^a^	Cola de caballo, caballo chupa	Native	Herb	Branch	Anti-inflammatory, antiseptic, diuretic, febrifuge, hepatic, hypotensive	NE	FT031t
*Equisetum giganteum* L.^a^	Cola de caballo, chupa caballo	Native	Herb	Plant without root	Anti-inflammatory, antiseptic, depurative, diuretic, hepatic	NE	FT1009
Fabaceae							
*Indigofera suffruticosa* Mill*.* ^a^	Tintanil	Native	Shrub	Branch, flower	Anti-inflammatory, tonic	NE	FT21t
Gentianaceae							
*Centaurium erythraea* Rafn	Canchalagua	Introduced	Herb	Whole plant	Antiflu, blood circulation, hypolipemiant, tonic	NE	FT1016
Geraniaceae							
*Erodium cf. cicutarium* (L.) L'Hér. ex Aiton	Agujilla	Introduced	Herb	Branch	Analgesic, anti-inflammatory, tonic	NE	FTE001MC
*Pelargonium graveolens* L'Hér.ex. Aiton	Esencia de rosa, malva esencia	Introduced	Herb	Leaf	Analgesic, anti-inflammatory, antidiarrheal, carminative, diuretic	NE	FT1258
*Pelargonium odoratissimum* (L.) L´Hér.	Malva olorosa	Introduced	Herb	Branch	Analgesic, anti-inflammatory, carminative, tonic	NE	FT016t
*Pelargonium zonale* (L.) L'Hér.	Geranio, geranio rojo	Introduced	Herb	Petal	Antiseptic, anti-inflammatory, astringent, diuretic, vulnerary	NE	FT17t
Lamiaceae							
*Melissa officinalis* L.	Toronjil	Introduced	Herb	Branch	Analgesic, anti-inflammatory, antispasmodic, cardiotonic, digestive, sedative	NE	FT45MPAT
*Mentha* x *piperita* L.	Hierba buena, menta	Introduced	Herb	Leaf	Analgesic, antidiarrheal, antiflu,, anti-inflammatory, antitussive, carminative, digestive, tonic	NE	FT1261
*Mentha spicata* L.	Hierba buena, menta, menta negra	Introduced	Herb	Leaf	Analgesic, anti-inflammatory, antiflu, antidiarrheal, antitussive, carminative, digestive, febrifuge, tonic	LC	FT1260
*Ocimum basilicum* L.	Albahaca, albahaca de sal, albahaca negra	Introduced	Herb	Leaf	Analgesic, antidiarrheal, antitussive, carminative, hepatic, digestive, diuretic, sedative, stomachache, tonic	NE	FT46TMPA
*Ocimum campechianum* Mill.	Albahaca, albahaca blanca, albahaca de dulce	Native	Herb	Leaf	Analgesic, blood pressure, carminative, digestive, restorative, sedative, stomachache, tonic	NE	FT46aMPA
Linaceae							
*Linum usitatissimum* L.	Linaza	Introduced	Herb	Seed	Anti-inflammatory, digestive, diuretic, hepatic, stomachache	NE	FT47TMPA
Malvaceae							
*Alcea rosea* L.	Malva goma, malva rosa, malvón	Introduced	Herb	Flower	Analgesic, anti-inflammatory, depurative, diuretic, tonic	NE	HUTPL891
*Malva arborea* (L.) Webb & Berthel.	Malva altea, malva blanca	Introduced	Shrub	Flower, leaf	Anti-inflammatory, depurative, tonic	NE	FT042MC
*Malva parviflora L.*	Malva alta, malva blanca	Introduced	Herb	Flower, leaf	Anti-inflammatory, depurative, hepatic, tonic	NE	FT015MCE
Myrtaceae							
*Myrcianthes hallii* (O. Berg) McVaugh	Arrayán	Native	Tree	Leaf	Analgesic, antiflu, anti-inflammatory, antispasmodic, hepatic, restorative	NE	FT215G
Onagraceae							
*Fuchsia harlingii* Munz	Pena pena, pena pena de la montaña	Endemic	Shrub	Flower	Anti-inflammatory, sedative	VU	HUTPL5784
*Fuchsia hybrida* Hort. T. ex Siebert & Voss	Pena pena grande, pena pena roja	Introduced	Shrub	Flower	Anti-inflammatory, antiflu, cardiotonic, sedative, stomachache	NE	FT1262
*Fuchsia loxensis* Kunth	Pena pena rosada	Endemic	Shrub	Flower	Cardiotonic, febrifuge, sedative	LC	FT1158
*Fuchsia magellanica* Lam.	Pena pena morada	Introduced	Shrub	Flower	Sedative	NE	FT0147
*Ludwigia* nervosa (Poir.) H. Hara	Flor de reina, mejorana de huerta	Native	Shrub	Flower	Anti-inflammatory, sedative	NE	FT194
*Oenothera rosea* L'Her. ex Aiton	Shullu	Native	Herb	Plant without root	Anti-inflammatory, digestive, diuretic, hepatic	NE	FT53MPAT
Orchidaceae							
*Epidendrum jamiesonis* Rchb. f.^a^	Flor de Cristo, maywa	Native	Epiphyte	Flower	Anti-inflammatory, sedative, diuretic, hepatic	A II	FT1063
Oxalidaceae							
*Oxalis corniculata* L.^a^	Chulco, chulku	Native	Herb	Branch	Antidiarrheal, anti-inflammatory, diuretic, hepatic, restorative	NE	FT1022
Piperaceae							
*Peperomia galioides* Kunth	Congona de cerro, sacha congona	Native	Herb	Plant without root	Analgesic, cardiotonic, diuretic, sedative, stomachache, tonic	NE	FT124SAR
*Peperomia ilaloensis* Sodiro	Congona de castilla, congona negra	Native	Herb	Plant without root	Analgesic, sedative	NE	FT01t
*Peperomia inaequalifolia* Ruiz & Pav.	Congona, congona grande	Native	Herb	Branch	Analgesic, cardiotonic, diuretic, hepatic, sedative	NE	FT1197
*Piper crassinervium* Kunth	Waviduca de dulce	Native	Shrub	Leaf	Analgesic, antiseptic, stomachache	NE	FT237
Plantaginaceae							
*Plantago major* L.	Llantén	Introduced	Herb	Plant without root	Anti-inflammatory, diuretic, hepatic, stomachache, vulnerary	LC	FT13t
Poaceae							
*Cymbopogon citratus* (DC.) Stapf	Hierba luisa, paja luisa	Introduced	Herb	Leaf	Analgesic, cardiotonic, digestive, diuretic, hypertensive, sedative, stomachache	NE	FT011t
*Cynodon dactylon* (L.) Pers.	Grama dulce	Introduced	Herb	Young branch	Analgesic, anti-inflammatory, antiseptic, diuretic, hepatic	NE	FT008MCE
*Hordeum vulgare* L.	Cebada	Introduced	Herb	Seed	Anti-inflammatory, digestive, sedative	NE	MCE017
Polypodiaceae							
*Niphidium crassifolium* (L.) Lellinger^a^	Calawala	Native	Epiphyte	Root	Anti-inflammatory, diuretic, hepatic	NE	FT40T
Proteaceae							
*Oreocallis grandiflora* (Lam.) R. Br.^a^	Cucharillo, gañil	Native	Tree	Flower	Anti-inflammatory, digestive, diuretic, hepatic, hypoglicemic agent, vulnerary	NE	FT04t
Pteridaceae							
*Pityrogramma ebenea* (L.) Proctor^a^	Doradilla plateada, luna plateada	Native	Herb	Leaf	Restorative, stomachache, tonic	NE	FT014
Rosaceae							
*Alchemilla aphanoides* Mutis ex L. f.	Saucillo	Native	Herb	Branch	Analgesic	NE	FT007
*Duchesnea indica* (Andrews) Teschem.	Fresa salvaje	Introduced	Herb	Leaf	Depurative	NE	FT0019t
*Rosa cymosa* Tratt.	Rosa, rosa de castilla	Introduced	Shrub	Petal	Anti-inflammatory, antiseptic, digestive, sedative, tonic	NE	FT274
*Sanguisorba minor* subsp. *muricata* (Bonnier & Layens) Briq.	Pimpinela	Introduced	Herb	Flower, leaf	Astringent, cardiotonic, depurative, hemostatic, hypoglycemic agent, hypertensive, sedative, tonic	NE	FT014MZ
Rutaceae							
*Citrus* x *junos* Siebold ex Tanaka	Naranja agria, naranjo	Introduced	Tree	Leaf	Analgesic, anti-inflammatory, digestive, sedative, stomachache	NE	FT007t
Solanaceae							
*Solanum americanum* Mill*.*	Hierba mora, mortiño	Native	Herb	Leaf	Analgesic, anti-inflammatory, digestive, febrifuge, sedative, stomachache	NE	FT36t
*Streptosolen jamesonii* (Benth.) Miers^a^	Flor de quinde, flor del sol, jaboncillo	Native	Shrub	Flower	Anti-inflammatory	NE	FT12t
Tiliaceae							
*Triumfetta semitriloba* Jacq.^a^	Abrojo, cadillo, mostrante	Native	Shrub	Flower, leaf	Analgesic, anti-inflammatory, astringent, diuretic, febrifuge	NE	FT39T
Verbenaceae							
*Aloysia triphylla* Royle	Cedrón	Native	Shrub	Flower, leaf	Analgesic, antispasmodic, blood pressure, cardiotonic, carminative, digestive, hepatic, hypoglycemic agent, restorative, sedative, stomachache	NE	FT1265
*Phyla scaberrima* (A. Juss. ex Pers.) Moldenke	Buscapina, novalgina	Introduced	Shrub	Plant without root	Analgesic, carminative, digestive, stomachache	NE	FT1240
Violaceae							
*Viola odorata* L.	Violeta, violeta de jardín	Introduced	Herb	Flower	Antibronquitic, antiflu,, anti-inflammatory, antitussive, depurative, digestive, hoarseness, vulnerary	NE	FT1266
*Viola tricolor* L.	Pensamiento	Introduced	Herb	Flower	Analgesic, antidiarrheal, antiflu, anti-inflammatory, antiseptic, diuretic, febrifuge, hoarseness	NE	FT1267
Zingiberaceae							
*Hedychium coronarium* J. Koening	Caña agria	Introduced	Herb	Stem	Anti-inflammatory, antiseptic, diuretic	NE	FT002t

Conservation status: *LC* = Least concern, *NE* = Not evaluated, *VU = * Vulnerable; A II = CITES Appendix II

^a^Wild medicinal plant species collected in nature by women

**Fig. 2:**
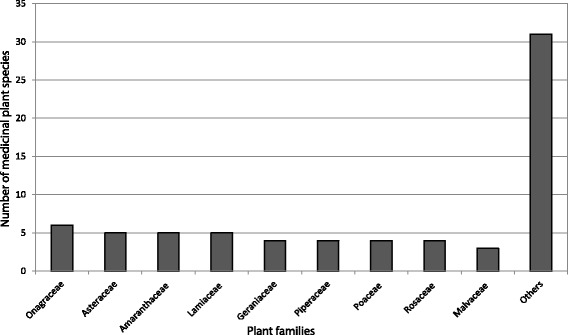
Dominant medicinal plant families used to prepare “horchata” drink in the Loja province, Southern Ecuador

Further analyses on the plant families show that Onagraceae has six species, being the dominant family. Asteraceae, Amaranthaceae and Lamiaceae are represented by five species each, followed by Geraniaceae, Piperaceae, Poaceae and Rosaceae with four species each, and Malvaceae with three species (Fig. 2). Likewise, six families have two species each, and 19 families contain a single species (Fig. 2). The plant families registered in the present study are the same as those reported in the three previous studies on “horchata” in Southern Ecuador [17, 21, 27]. This is an indication that there is a core group of 16 medicinal plant species in the recipe for the preparation of the “horchata” drink that provides aroma, color and flavor.

Each bunch of medicinal plants sold to prepare “horchata” can vary from 16 to 32 species, depending on their spatial and temporal availability. The presence of the remaining 39 species is conditional on the seasonal stock and the quantities of plant material during the year. The most frequently used species for the preparation of this drink coincide with those reported in the three previous studies on “horchata” [17, 21, 27], and are often those which provide aroma (*Alcea rosea, Aloysia triphylla, Cymbopogon citratus,* and *Mentha* x *piperita* or *Menta spicata*), color (*Aerva sanguinolenta*, *Amaranthus hybridus* or *Iresine diffusa*), and flavor (*Borago officinalis*, *Citrus* x *junos*, *Dianthus caryophyllus, Equisetum bogotense*, *Foeniculum vulgare*, *Fuchsia hybrida*, *Matricaria recutita, Melissa officinalis, Pelargonium graveolens* or *P. odoratissimum*, *Plantago major*, and *Triumfetta semitriloba*)*.*

### Medicinal plants: habits, geographic range, and conservation status

The most frequent habits of medicinal plants were herbs with 50 species, followed by shrubs (14 spp.), trees (4 spp.), epiphytes (2 spp.), and a vine (1 sp.). The high frequency of the herb habit reported in this study is similar to others in the Andean highlands, such as Bolivia [62], Peru [63] and Ecuador [30]. This similarity is likely due to the random occurrence of herbaceous species in nature, trends in plant species diversity, and geographic endemism. Likewise, participant observation revealed how medicinal herbs are easily handled by women. Because of their short life cycle, these plants can be effortlessly propagated in homegardens and at certain natural vegetation patches, such as roadsides, ravines, riversides, and buffer zones at the Andean paramo.

Among the 71 medicinal plant species sold to prepare the “horchata” drink, 38 are introduced, 29 are native and three are endemic. At the 31 traditional markets, these 38 introduced species of medicinal plants are locally produced in homegardens, but have originated in different regions of the World [21]. Of the 29 plant species native to Ecuador, 45% are cultivated in homegardens, 35% are harvested by women from mountain forest, cloud forest, scrub vegetation, and Andean paramo, while the remaining 20% can be found at any of the aforementioned habitats.

It is important to emphasize that of the 71 medicinal plants species, only three are endemic to the Andean highlands of Ecuador. These correspond to two plant families Asclepiadaceae, characterized by the vine *Orthosia ellemannii*, and Onagraceae represented by two shrubs *Fuchsia harlingii* and *Fuchsia loxensis*. These three plant species are wild and difficult to find in nature; additionally, they are included in the red book of endemic plants of Ecuador [45]. When considering their geographic status and distribution in nature, it becomes evident that these three species are uncommon, and that is the reason why they are seldom found in traditional markets as part of the bunch sold to prepare “horchata”.

There are ten medicinal plant species sold in over half of the 31 traditional markets, eight of which are cultivated in homegardens and two are wild species. The former are *Aerva sanguinolenta*, *Aloysia triphylla, Amaranthus hybridus, Malva arborea, Matricaria recutita, Pelargonium graveolens, Plantago major* and *Oenothera rosea.* The latter are *Equisetum bogotense* and *Oreocallis grandiflora.* The remarkable presence of these plant species at traditional markets is a consequence of their local accessibility. Firstly, explained by their widespread distribution in the Loja province, and secondly, by the available quantities of plant material throughout most of the year. The ability to access and use these ten species strengthens the relationship between women vendors and buyers, as the latter often satisfy their need for consumption.

When the conservation status of the 71 medicinal plant species was revised in TROPICOS database [38], the Red List of the IUCN [44], and the red book of endemic plants of Ecuador [45], 65 species are not evaluated (NE) and are subjects for further studies. It is important to evaluate all the 71 plant species (Table 2), emphasizing on the native and endemic; also, it should be consider their sustainable harvest and role in market transactions. Of the 38 introduced plant species, 5% are categorized under Least Concern (LC), which are *Mentha spicata* and *Plantago major*. Of the 29 native plant species, 3% is registered in the CITES Appendix II, which is *Epidendrum jamiesonis*. All three endemic plant species are in a category of endangerment, which are O*rthosia ellemannii, Fuchsia harlingii,* and *Fuchsia loxensis*.

O*rthosia ellemannii* (Asclepiadaceae) and *Fuchsia harlingii* (Onagraceae) were registered as Vulnerable (VU), which is the category with the highest risk of endangerment. *Mentha spicata* (Lamiaceae), *Fuchsia loxensis* (Onagraceae) and *Plantago major* (Plantaginaceae) are under the category LC, which is a lower category of risk. The orchid *Epidendrum jamiesonis* (Orchidaceae) is included in the species list of CITES Appendix II. The formerly mentioned plant species represent 9% of the total, and their conservation requires research on propagation, wild harvest, ecofriendly conservation and biocommerce policies.

### Vernacular names of medicinal plant species

A total of 118 vernacular names were recorded for the 71 medicinal plant species, 31 of which (44%) have at least one name, 32 (45%) have two names, 6 (8%) have three names, and 2 (3% have four names. Spanish is the predominant language with 94 (80%) vernacular names, followed by the Kichwa language from the Andean highlands with 24 (20%). The given name for the whole plant individual is identical to its morphological structures. A representative example in the family Onagraceae is the *Fuchsia* genus, all its species are recognized by “horchateras” and women vendors at traditional markets with the generic name of “pena pena”, and the specific epithet varies according to the color and size of the flower.

Among the present study and the three previously made at Loja province by Arguello and Aguilar [27], Cerón [17], and Villamagua Vergara [21], there was a 95% concordance in the use of vernacular names of the medicinal plants to prepare “horchata”. It is important to highlight that the plant species that provide color, aroma and flavor for this drink are recognized with the same vernacular name by the 185 vendors. This last particular ethnobotanical aspect shows that there is a flux of ancestral wisdom in regards to the vernacular names of the medicinal plants used to prepare “horchata”.

### Medicinal plant species: morphological structures and therapeutic use

The analysis reveals that there are 11 kinds of morphological plant structures that are sold for the preparation of the “horchata” drink as medicinal materials (Table 2). Leaves are the most frequently used plant morphological structures (23%), followed by flowers (22%), branches (14%), entire plants without roots (12%), and petals (5%). Also, the study found that some specific morphological structures such as inflorescence, root, seed, shoot, stem, and whole plant are sold less frequently.

The women market vendors mention 32 kinds of therapeutic uses for the 71 registered medicinal plant species, all are sold as fresh plant material at the 31 traditional markets. The 66% of medicinal plant species are used as anti-inflammatories, 51% as analgesics, 42% as diuretics, and between 28 and 37% as sedatives, tonics, digestives, hepatics and palliatives for stomachache. Between 1 and 24% of the medicinal plant species are applied for 24 therapeutic uses. Half of the medicinal plant species sold as anti-inflammatories are also analgesics; therefore it is important to conduct phytochemical studies to understand the dual effect and discover possible adverse secondary outcomes.

Indigenous people in Loja province believe that the use of certain plant morphological structures and their characteristics are indicative of their therapeutic use. One relevant case is the inflorescence of *Amaranthus caudatus*, which has a dusky red color and is used to improve blood circulation and as an emmenagogue. For applying the therapeutic uses of medicinal plants, local indigenous people associate the aroma, color, flavor, shape, or texture of some morphological structures or whole plant individuals to a particular fluid or organ in the human body. In this research, the participant observation allowed for a level of understanding of how, through local cultural beliefs and cosmovision, the plant world used for the preparation of the “horchata” drink is related to the human body and its feelings.

### Fidelity level index and cultural importance of medicinal plants

The FL index measured the agreement on the therapeutic use of certain medicinal plant species among women market vendors who sold bunches to prepare “horchata”. When calculating the FL index for each of the 71 medicinal plant species mentioned by women vendors, the cultural importance was determined according to the number of times its therapeutic use was mentioned by each woman vendor. The FL index was also useful for underlining the most relevant plant species sold in each “horchata” bunch.

There are 20 culturally important medicinal plants species determined by the FL index, and sold at the 31 traditional markets in Loja province as part of the bunches for preparing “horchata” (Table 3). This 20 medicinal plant species stand out due to their application for 25 therapeutic uses according to 34 or more women market vendors. The species with the largest FL index is *Amaranthus hybridus* with 96.3%, and the other 19 species have FL values between 46.1 and 82.1% (Table 3).

**Table 3:** Fidelity level of medicinal plant species and therapeutic use in the Loja province

Number	Medicinal plant species	Therapeutic use	Ip	Iu	*FL value (%)
1	*Amaranthus hybridus* L.	Anti-inflammatory, diuretic, emmenagogue, tonic, vulnerary	78	81	96.3
2	*Melissa officinalis* L.	Analgesic, anti-inflammatory, antispasmodic, cardiotonic, digestive, sedative	78	95	82.1
3	*Equisetum bogotense* Kunth*	Anti-inflammatory, antiseptic, diuretic, febrifuge, hepatic, hypotensive	86	106	81.1
4	*Foeniculum vulgare* Mill.	Analgesic, carminative, digestive, diuretic, hepatic, stomachache, tonic	42	55	76.4
5	*Plantago major* L.	Anti-inflammatory, diuretic, hepatic, stomachache, vulnerary	91	121	75.2
6	*Matricaria recutita* L.	Analgesic, antidiarrheal, anti-inflammatory, carminative, digestive, sedative, stomachache	76	105	72.4
7	*Triumfetta semitriloba* Jacq.*	Analgesic, anti-inflammatory, astringent, diuretic, febrifuge	52	74	70.3
8	*Pelargonium odoratissimum* (L.) L'Hér.	Analgesic, anti-inflammatory, carminative, tonic	34	49	69.4
9	*Iresine difusa* Humb. & Bonpl. ex Willd.	Analgesic, antiflu, anti-inflammatory, diuretic, hepatic, tonic	36	52	69.2
10	*Pelargonium graveolens* L'Hér.ex. Aiton	Analgesic, anti-inflammatory, antidiarrheal, carminative, diuretic	49	71	69.1
11	*Dianthus caryophyllus* L.	Analgesic, anti-inflammatory, cardiotonic, restorative, sedative	70	104	67.3
12	*Citrus* x *junos* Siebold ex Tanaka	Analgesic, anti-inflammatory, digestive, sedative, stomachache	42	63	66.7
13	*Borago officinalis* L.	Analgesic, antidiarrheal, antiflu, anti-inflammatory, antitussive, emmenagogue, febrifuge	79	119	66.4
14	*Cymbopogon citratus* (DC.) Stapf	Analgesic, cardiotonic, digestive, diuretic, hypertensive, sedative, stomachache	60	98	61.2
15	*Mentha* x *piperita* L.	Analgesic, antidiarrheal, antiflu,, anti-inflammatory, antitussive, carminative, digestive, tonic	48	79	60.8
16	*Fuchsia hybrida* Hort. T. ex Siebert & Voss	Anti-inflammatory, antiflu, cardiotonic, sedative, stomachache	36	62	58.1
17	*Alcea rosea* L.	Analgesic, anti-inflammatory, depurative, diuretic, tonic	48	87	55.2
18	*Aloysia triphylla* Royle	Analgesic, antispasmodic, blood pressure, cardiotonic, carminative, digestive, hepatic, hypoglycemic agent, restorative, sedative, stomachache	53	99	53.5
19	*Aerva sanguinolenta* (L.) Blume	Analgesic, anti-inflammatory, antiseptic, diuretic, emmenagogue, restorative, stomachache	98	185	52.9
20	*Mentha spicata* L.	Analgesic, anti-inflammatory, antiflu, antidiarrheal, antitussive, carminative, digestive, febrifuge, tonic	41	89	46.1

Ip = Number of women market vendors who independently cited the importance of a specific therapeutic use

Iu = Total number of women market vendors

^*^FL value (%) = Fidelity level value percentage (0 = the least, 100 = the highest agreement)

A medicinal plant species with a FL index of 100% would always have reported uses for the same therapeutic treatment. The FL value for *Amaranthus hybridus* (96.3%) indicates that it is consistently used as an antiflu, anti-inflammatory, diuretic, emmenagogue, tonic, and vulnerary. This high value for *A. hybridus* can be explained by the women vendors’ perception of its considerable therapeutic efficacy. Cultural persistence is possible when local ancestral wisdom are linked to the medicinal use of *A. hybridus*; particularly, when its herbal infusion alleviates the symptoms of six ailments mentioned before. In addition, the fuchsia tonality of its infusion makes people feel invigorated.

The seven medicinal plant species with a FL > 70% are *Amaranthus hybridus*, *Melissa officinalis*, *Equisetum bogotense*, *Foeniculum vulgare*, *Plantago major*, *Matricaria recutita*, and *Triumfetta semitriloba*. Since these plant species are frequently present in “horchata” bunches, biochemical analyses are recommended for establishing their efficacy, safety, and reliability.

In the long-term, new products as nutraceuticals, phytopharmaceuticals, and pharmafoods could be developed by including a single or multiple plant species. The evaluation of these seven species through laboratory testing on phytochemistry, bromatology, pharmacology, bioactivity essays, and toxicity may lead to the use of these plants in traditional medicine, biocommerce and fair trade.

Ultimately, the government should procure an equitable distribution of the financial benefits that could result from the placement of “horchata” in international markets or if bioactive compounds are found after successful research and bioprospecting efforts. This will only be possible if the small local enterprises establish and/or retain their internal social structure and practice sustainable management plans for the wild plant species used in the “horchata”.

When comparing the present research with Tinitana et al. [30] conducted at traditional markets in Southern Ecuador, both studies show a strong consensus of the medicinal plants used to treat gastrointestinal afflictions in the Loja province. This can be seen in four culturally important medicinal plant species with FL index values above 72.4%: *Aerva sanguinolenta*, *Borago officinalis*, *Matricaria recutita*, and *Melissa officinalis*. All this plants have morphological structures used to treat symptoms related to gastrointestinal and stomach disorders and are applied as analgesic, antidiarrheal, antispasmodic, carminative, and digestive.

### Factor of informant consensus of therapeutic uses and medicinal plants

The FIC index values were used to determine agreement among vendors on the therapeutic use of the medicinal plants (Table 4). The FIC values range between 0.50 and 1.00. Four therapeutic uses have FIC = 1.00. The remaining 28 therapeutic uses correspond to values of FIC between 0.50 and 0.99. The 11 therapeutic uses with a FIC > 0.90 are related to the treatment of symptoms involving the circulatory, digestive and respiratory systems.

**Table 4:** Factor of informant consensus for therapeutic uses of medicinal plant species in the Loja province

N°	Therapeutic uses of medicinal plants^**a**^	Number of medicinal plant species	Number of use citations by market vendors	FIC^b^
1	Antibronquitic	1	78	1.00
2	Cholagogue	1	93	1.00
3	Hemostatic	1	27	1.00
4	Stimulant	1	81	1.00
5	Blood circulation	2	115	0.99
6	Antispasmodic	4	178	0.98
7	Restorative	9	179	0.96
8	Vulnerary	7	137	0.96
9	Antidiarrheal	9	175	0.95
10	Astringent	3	39	0.95
11	Hoarseness	2	15	0.93
12	Tonic	11	137	0.93
13	Antiseptic	9	97	0.92
14	Cardiotonic	10	119	0.92
15	Blood pressure	2	12	0.91
16	Febrifuge	9	86	0.91
17	Digestive	21	182	0.89
18	Hepatic	21	184	0.89
19	Stomachache	20	164	0.88
20	Carminative	13	89	0.86
21	Sedative	26	173	0.86
22	Antiflu	17	95	0.83
23	Diuretic	30	169	0.83
24	Analgesic	36	185	0.81
25	Depurative	8	35	0.79
26	Antitussive	6	23	0.77
27	Hypertensive	2	5	0.75
28	Hypolipemiant	2	5	0.75
29	Emmenagogue	4	12	0.72
30	Anti-inflammatory	47	133	0.65
31	Hypoglycemic agent	2	3	0.50
32	Hypotensive	3	5	0.50

^a^ The therapeutic uses follow the categories proposed by Rios et al. [25] and were applied to the 71 medicinal plant species sold at 31 traditional markets at Loja province, Southern Ecuador

^b^ *FIC*= Factor of Informant Consensus

The therapeutic uses with a FIC = 1.00 correspond to four medicinal plant species commonly sold by vendors. Between 27 and 93 women are responsible for the following one-to-one consensus: 1) antibronquitic, *Viola odorata*; 2) cholagogue, *Taraxacum officinale*; 3) stimulant, *Amaranthus caudatus*; and 4) hemostatic, *Sanguisorba minor* subsp. *muricata*.

The species therapeutic uses for blood circulation, blood pressure, cardiotonic, depurative, restorative, sedative, and tonic purposes have relatively large FIC values, ranging between 0.91 and 0.99. It is notable that these seven therapeutic uses are related to the circulatory and nervous systems, since the intense fuchsia tonality of “horchata” is culturally associated with improving blood quality, spiritual strength, and emotional wellbeing. When the present study FIC values were compared to those reported by Tinitana et al. [30], a consensus was found in the medicinal purposes for *Aloysia triphylla, Amaranthus hybridus,* and *Melissa officinalis.*

The species therapeutic uses for blood circulation, blood pressure, cardiotonic, depurative, restorative, sedative, and tonic purposes have relatively large FIC values, ranging between 0.91 and 0.99. It is notable that these seven therapeutic uses are related to the circulatory and nervous systems, since the intense fuchsia tonality of “horchata” is culturally associated with improving blood quality, spiritual strength and emotional wellbeing. When the present study FIC values were compared to those reported by Tinitana et al. [30], a consensus was found in the medicinal purposes for *Aloysia triphylla, Amaranthus hybridus,* and *Melissa officinalis.*

The plants therapeutic uses for analgesic, antidiarrheal, anti-inflammatory, antiseptic, antispasmodic, astringent, carminative, digestive, diuretic, hepatic, and stomachache purposes have FIC values ranging between 0.81 and 0.98. These eleven therapeutic uses are related to the digestive and genitourinary systems, which is relevant in the Loja province given that there is a high incidence of gastrointestinal diseases, including stomach cancer [64–70]. When comparing these FIC values to those reported by Tinitana et al. [30], there is a strong agreement on the use of *Matricaria recutita* among women vendors.

The therapeutic uses of antiflu, antitussive, febrifuge, hoarseness, and vulnerary are included in a range of FIC values from 0.77 to 0.96. These five therapeutic uses are related to the respiratory system, which includes a variety of diseases that are manifested in children and the elderly of Loja province [71]. When the FIC values were contrasted to those in Tinitana et al. [30], there is a strong consensus in the medicinal purpose of *Borago officinalis* and *Citrus* x *junos*.

The lowest FIC values (0.50 to 0.79) represent the least agreement among women vendors for medicinal plants species that are sold to treat the symptoms of five therapeutic uses, which are: hypoglycemic agent, hypolipemiant, hypertensive, emmenagogue, and hypotensive. These low FIC values could be due to the lack of flux in ancestral wisdom among women vendors, since they live in different settlements and sell plants at diverse traditional markets.

### Cluster analysis of bunches sold to prepare the “horchata” drink

**Fig. 3:**
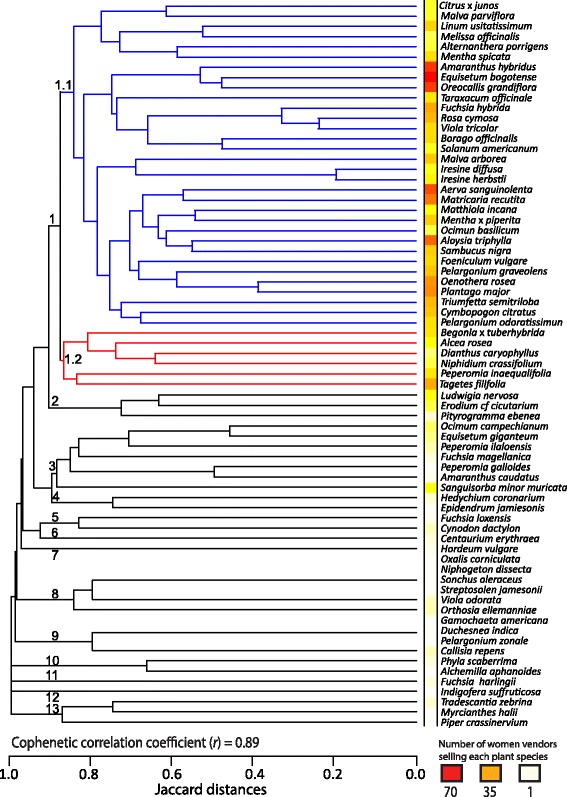
Medicinal plant species similarity among bunches sold to prepare “horchata” drink at traditional markets in the Loja province, Southern Ecuador

The present study is the first in Ecuador to apply as an analytical tool a cluster analysis to study the composition of “horchata” bunches sold at the 31 traditional markets. This statistical tool grouped medicinal plant species by their similarities in the bunch sold by women vendors. The dendrogram has a cophenetic correlation of *r* = 0.89, showing a strong correspondence between the original data matrix and the resulting clustering pattern (Fig. 3). There are 13 clusters or “horchata” bunches with a Jaccard distance value of 0.9, where cluster 1 stands out with 38 most frequently sold medicinal plant species (Fig. 3).

In cluster 1, the 38 plant species represent 45% of the total number. These species are divided into two sub-clusters (Fig. 3). The sub-cluster 1.1 contains 32 medicinal plants, including the 16 species that represent the core group that provides aroma, color and flavor to the drink. The sub-cluster 1.2 has six medicinal plants that replace the species in sub-cluster 1.1. Their role in replacing the other species depend on their seasonal stock and yearly quantities. For instance, *Alcea rosea* replaces *Aloysia triphylla, Cymbopogon citratus, Mentha* x *piperita* or *Menta spicata* when the latter are not available.

Of the 38 species that form cluster 1, a total of 97% are mentioned in the surveys made in the Loja province by Arguello and Aguilar [27], Cerón [17], and Villamagua Vergara [21]. When the therapeutic uses of medicinal plants reported in the three previous surveys [17, 21, 27] are compared to the present study, 99% match on their applications in traditional medicine. A notable example is *Equisetum bogotense*, known by the vernacular name “cola de caballo” (horse tail), which is a wild medicinal plant species commonly used as a diuretic in Loja. Other two wild medicinal plant species in cluster 1, *Oreocallis grandiflora* and *Triumfetta semitriloba*, have the same therapeutic uses in the current and the three past studies on “horchata” [17, 21, 27].

The last 12 clusters contain 33 medicinal plant species. The availability of the 23 medicinal plants coming from homegardens depend on the agricultural calendar of “horchateras.” Thus, 15 species are cultivated and eight grow as weeds in cultivars or the surrounding areas. The geographical distribution (Table 2), seasonal stock and quantity throughout the year determine the presence of the 10 wild medicinal plants in the “horchata” bunches. The quantity available is dependent on two factors: when women have enough time to go to nature and collect plants, and when women find sufficient plant material for self-consumption and for sale.

When comparing the 10 wild medicinal plants present in the former 12 clusters with the reported species in the three previous surveys on “horchata” [17, 21, 27], it is remarkable that only *Equisetum giganteum* has been previously reported [19]. Therefore, the present study is the first to identify the presence of nine wild medicinal plant species as part of the bunches (Table 2). It is important to study the traditional formula, especially for each region in the Loja province, to prepare “horchata.” The formula can vary among localities and even within communities, particularly because of preparation secrets kept within families.

## Conclusions

The present study contributes to ethnobotany and ethnomedicine by reporting 71 medicinal plant species, which is the largest number reported up-to-date in surveys related to “horchata”; this underlines the cultural value of this herbal mixture infusion at the Loja province. “Horchata” is consumed by all social strata, which maintains traditional practices related to medicinal plants and people wellbeing. Tinitana et al. [30] made similar observations on “horchata” use in Southern Ecuador; where “horchateras” and women market vendors are key players for the cultural conservation and persistence of the “horchata” consumption.

Women are the principal stakeholders by caring and growing medicinal plant cultivars at their homegardens, selling bunches for “horchata”, and preparing this ancestral drink. In relation to ethnobotany, women preserve an ancestral tradition linked to the management of medicinal plants, which traverses oral history between mothers and daughters, grandmothers and grandchildren, aunts and nieces, and among other filial bonds related to the female line or matrilineality. Either a glass of cold “horchata” or a cup of hot “horchata” symbolizes a cultural tradition with different time periods. Because of its historical use, “horchata” could become a patrimonial heritage.

With the data collected, it became clear that all medicinal plants are sold fresh and that bunch mixes need to have an analgesic, anti-inflammatory, digestive, diuretic, hepatic and sedative effect. The herbal mixtures are linked to the six formerly mentioned therapeutic uses, and it is important to determine if a bunch functions as a palliative or curative. However, it must be taken into account that there is a high incidence of stomach cancer and gastrointestinal afflictions in the Loja province [70, 71].

It is crucial to understand if local people self-medicate by consuming “horchata” on a daily basis in the Loja province. Experiments are required to determine the appropriate doses or posology of this herbal mixture infusion. There is a necessity to implement policies for medicinal plants trade and quality in traditional markets, especially to determine if they come from organic cultivars in homegardens or contain agrochemicals. The reasons could range from the lower cost of the drink, confidence in traditional medicine, psychosomatic effects, sociocultural environment, family tradition, and regional heritage. Furthermore, studies are needed to establish the consequence of “horchata” in human health, mostly when it is consumed almost daily and the effect of each medicinal plant at the somatic and/or psychosomatic level remains unknown.

This survey reports the presence of 13 wild medicinal plant species (Table 2), for which it is necessary further research on the overharvesting and trade demand of their morphological structures; particularly, when these are flowers or fruits that are reproductive organs and are essential to propagation. The trade demand of medicinal plants species used for “horchata” could increase over the next few years, especially if this drink becomes even more popular and increased consumption could endanger the natural populations of wild plant species.

It is important to consider all possible implications to the conservation of wild medicinal plant species that are frequently used for the “horchata” drink, because sustainable management of these species will be necessary to avoid their extinction. The implementation of actions for the in situ recovery of wild medicinal plant populations in Loja is urgently needed to safeguard their survival. Ethnobotanical research should register all the wild plants used, especially those more frequently included as part of “horchata”, and revise them in all available red lists at a local, regional and global level.

The traditional use of “horchata” has extended to other localities in Ecuador, and even to other countries. This drink has become so popular that its medicinal plants are sold dehydrated in plastic bags or pulverized in sachets at supermarket chains, retail stores, and at some fair trade stores in the United States and Europe. When “horchata” is sold as dried plant material, hydration in hot water is necessary. Chemical analyses are required to determine if it has the same effects as in the fresh form. Scientific studies are needed to understand any undesired or secondary effects of the medicinal plant species used in “horchata”. Likewise, it is also necessary to determine which formula for “horchata” is the optimal for its effects on human health and wellbeing, especially in regards to which combination of medicinal plant species are used and how much plant material is required.

The combination of the FL and FIC index results, the cluster analysis, and the field observations reveal collective agreement held by 185 market vendors in the Loja province, mainly related to a 16 plant species and to their therapeutic use. This medicinal plants core group is always present in “horchata”, being essential for its organoleptic qualities and therapeutic uses. The high values of FL (46.1–96.3) and FIC (1.00) indices pointed out 20 culturally important plants species that treat ailments related to the circulatory, digestive, nervous, and respiratory systems. Bioactivity and bioassays analyses are needed to determine the real benefits of the plants core group and if they potential could be a future key for biomedicine.

The grouping pattern inferred by the cluster analysis is the result of the similarities among the medicinal plant species used to prepare “horchata”, and depended on which plant species were present in the bunches (Fig. 3). When relating the results obtained by participant observation and cluster analysis at the 12 established markets and 19 open markets, it was evident that the mix of medicinal plants sold depended on the vendor´s place of origin. Thus, each traditional market is characterized by its own particular assortment of “horchata” bunches. The type of bunches sold depended on where they were offered in Loja, and the distance between rural areas and cities or towns where medicinal plants were sold.

Cluster 1 grouped together 38 medicinal plant species that are the most frequently used and sold to prepare “horchata.” There is a cultural consensus in the Loja province with regards to the traditional formula (Fig. 3). When comparing the 38 medicinal plant species in cluster 1 with the remaining 33 species in the other 12 clusters, the latter are subject to be included in the “horchata” bunches depending on cultural, social and environmental circumstances (Fig. 3). The principal reasons why medicinal plant species were included in a bunch corresponded to their geographic distribution, adaptation according to their life form, availability during summer and winter, and for this last decade to the effect of climate change. An example is when there is an out-of-season frost, which causes damages to cultivars and affects plant populations in nature by reducing the number of individuals.

The presence of the 13 clusters suggests that it is necessary to further study the traditional formula for “horchata” according to the different regions in Loja, as this can vary due to the presence or absence of the 71 medicinal plant species recorded to date (Fig. 3). The “horchateras” and/or women vendors make the “horchata” bunches according to the availability of plants at homegardens and ecosystems with natural vegetation near their settlements. Additionally, the link between the geographic and cultural origin of “horchateras” with the plant diversity sold in each “horchata” bunch must be studied. This will allow the understanding on how the drink formula can vary within Loja, and increased knowledge on the local ethnoculinary and food patterns of Ecuadorian heritage.

This study advocates for “horchata” becoming a well-positioned, high-quality product in the fair trade industry. This could potentially improve the vendors’ quality of life. A “horchata” product with organic seals will increase the value of the product, improve income for the “horchateras” and promote sustainable development in Ecuador. The act of positioning a marketable product creates opportunities for local subsistence and the empowerment of “horchateras”. Therefore, it is important to contribute to a new and more inclusive regional agenda, as it will serve to integrate rural production with urban customers from local levels, and reaching sub-national, national, regional and global stakeholders.

Ecuador’s challenge is to build bridges between traditional cultivation and wild harvest with urban consumption patterns. This will happen in the future by creating alternative production philosophies with equal rights and opportunities. This is the case of “horchateras” as women who use, protect, conserve, restore and promote ecosystems of medicinal plant species in homegardens and nature. This assertion coincides with Voeks [72], Dembélé et al. [73], and Hunde et al. [74], who affirm that women discern the cultural value that medicinal plants have, as their family role empowers them to become reservoirs of important wisdom. The ancestral practices of women are linked to the sustainable management of useful plants and minimization of natural impact, because their subsistence is grounded on rational consumption and an environmentally friendly way of life.
